# Interaction of Vitamin D with Peptide Hormones with Emphasis on Parathyroid Hormone, FGF23, and the Renin-Angiotensin-Aldosterone System

**DOI:** 10.3390/nu14235186

**Published:** 2022-12-06

**Authors:** Nejla Latic, Reinhold G. Erben

**Affiliations:** Department of Biomedical Sciences, University of Veterinary Medicine, 1210 Vienna, Austria

**Keywords:** vitamin D, vitamin D metabolism, fibroblast growth factor-23, klotho, parathyroid hormone, 1α-hydroxylase, RAAS

## Abstract

The seminal discoveries that parathyroid hormone (PTH) and fibroblast growth factor 23 (FGF23) are major endocrine regulators of vitamin D metabolism led to a significant improvement in our understanding of the pivotal roles of peptide hormones and small proteohormones in the crosstalk between different organs, regulating vitamin D metabolism. The interaction of vitamin D, FGF23 and PTH in the kidney is essential for maintaining mineral homeostasis. The proteohormone FGF23 is mainly secreted from osteoblasts and osteoclasts in the bone. FGF23 acts on proximal renal tubules to decrease production of the active form of vitamin D (1,25(OH)_2_D) by downregulating transcription of 1α-hydroxylase (*CYP27B1*), and by activating transcription of the key enzyme responsible for vitamin D degradation, 24-hydroxylase (*CYP24A1*). Conversely, the peptide hormone PTH stimulates 1,25(OH)_2_D renal production by upregulating the expression of 1α-hydroxylase and downregulating that of 24-hydroxylase. The circulating concentration of 1,25(OH)_2_D is a positive regulator of FGF23 secretion in the bone, and a negative regulator of PTH secretion from the parathyroid gland, forming feedback loops between kidney and bone, and between kidney and parathyroid gland, respectively. In recent years, it has become clear that vitamin D signaling has important functions beyond mineral metabolism. Observation of seasonal variations in blood pressure and the subsequent identification of vitamin D receptor (VDR) and 1α-hydroxylase in non-renal tissues such as cardiomyocytes, endothelial and smooth muscle cells, suggested that vitamin D may play a role in maintaining cardiovascular health. Indeed, observational studies in humans have found an association between vitamin D deficiency and hypertension, left ventricular hypertrophy and heart failure, and experimental studies provided strong evidence for a role of vitamin D signaling in the regulation of cardiovascular function. One of the proposed mechanisms of action of vitamin D is that it functions as a negative regulator of the renin-angiotensin-aldosterone system (RAAS). This finding established a novel link between vitamin D and RAAS that was unexplored until then. During recent years, major progress has been made towards a more complete understanding of the mechanisms by which FGF23, PTH, and RAAS regulate vitamin D metabolism, especially at the genomic level. However, there are still major gaps in our knowledge that need to be filled by future research. The purpose of this review is to highlight our current understanding of the molecular mechanisms underlying the interaction between vitamin D, FGF23, PTH, and RAAS, and to discuss the role of these mechanisms in physiology and pathophysiology.

## 1. Introduction

Vitamin D was first described in the 1920s in attempts to find the cause of rickets that had reached epidemic proportions in industrial cities in Middle and Northern Europe at that time [1,2,3,4]. However, it would take almost another century after this seminal discovery until the key factors regulating vitamin D metabolism were found, and a more complete understanding of the vitamin D hormonal system had evolved. Triggered by the finding in the 1970s that parathyroid hormone (PTH) is a major endocrine regulator of vitamin D metabolism [5,6,7,8], and by the discovery of fibroblast growth factor-23 (FGF23) in the year 2000, it became clear that peptide hormones and small proteohormones play pivotal roles in the regulation of vitamin D metabolism by participating in the crosstalk between different organs. In addition, it was discovered in the year 2002 that renin secretion in the kidney is regulated by vitamin signaling, establishing a novel link between vitamin D and the renin-angiotensin-aldosterone system (RAAS) [9]. 

This review aims to summarize what we currently know about the interaction of the vitamin D hormonal system with peptide hormones and small proteohormones, focussing on PTH, FGF23, and the RAAS. 

## 2. Brief Overview of Vitamin D Metabolism

Vitamin D is a secosteroid derived from cholesterol in animals (cholecalciferol or vitamin D_3_), or from ergosterol in fungi and protozoa (ergocalciferol or vitamin D_2_). Both forms of vitamin D will be referred to as vitamin D in this review. Vitamin D is either taken up from dietary sources, or it is produced in the skin by UVB-mediated photochemical transformation of 7-dehydrocholesterol. Vitamin D itself is biologically inactive and needs to be metabolically activated by two hydroxylation steps occurring in the liver and the kidney. The canonical vitamin D activation pathway involves 25- and subsequent 1α-hydroxylation. The 25-hydroxylation step occurs in the liver, forming the most abundant circulating form of vitamin D, 25-hydroxyvitamin D (25(OH)D). There is only a little endocrine regulation of this step. In contrast, the 1α-hydroxylase (*CYP27B1*)-mediated synthesis of the vitamin D hormone, 1α,25-dihydroxyvitamin D (1,25(OH)_2_D) in proximal tubules of the kidney is strictly regulated [10]. The kidney is the major source of circulating 1,25(OH)_2_D under physiological conditions [11]. However, circulating 25(OH)D can also be converted into 1,25(OH)_2_D locally in cells expressing 1α-hydroxylase [12,13]. 1,25(OH)_2_D is the biologically active principle in the vitamin D hormonal system, regulating gene transcription through a nuclear receptor protein, the vitamin D receptor (VDR). It is now generally accepted that the VDR mediates all actions of the vitamin D endocrine system [14]. The VDR is ubiquitously expressed, and as a consequence vitamin D signaling has a major impact on gene regulatory networks in many cell types, regulating transcription of about 3% of the human genome [15]. Typically, the VDR-1,25(OH)_2_D complex binds to vitamin D response elements (VDREs) in regulatory regions of target genes as a heterodimer with the retinoid X receptor (RXR) [14]. 

The most important physiological function of 1,25(OH)_2_D is in the gut, stimulating intestinal absorption of calcium and phosphate. Therefore, intact vitamin D signaling is essential for bone and mineral homeostasis in most vertebrates [16]. To avoid hypercalcemia and hyperphosphatemia as a potential untoward consequence of excessive vitamin D signaling, the catabolism of 1,25(OH)_2_D is tightly regulated. Catabolism of 25(OH)D and 1,25(OH)_2_D in the kidney and all vitamin D target cells is mediated by 24-hydroxylase (*CYP24A1*), the key enzyme of the vitamin D inactivation pathway [17]. Several independent lines of evidence have shown that the regulation of *CYP24A1* is an important part of the homeostatic control of circulating and intracellular concentrations of 1,25(OH)_2_D [18,19] 

Although 25- and subsequent 1α-hydroxylation represent the predominant vitamin D activation pathway, other activation pathways exist, at least at a local level. It has been shown that the cytochrome p450 enzyme CYP11A1 is able to hydroxylate vitamin D_3_ at the C17, C20, C22, and C23 positions, giving rise to 20(OH)D_3_ and 22(OH)D_3_ as the major metabolites [20,21]. Vitamin D_2_ can be hydroxylated by CYP11A1 at the C17, C20, and C24 positions [20,21]. CYP11A1 is mainly found in adrenal glands, skin, and placenta. 20(OH)D_3_ and 22(OH)D_3_ are detectable in human serum, albeit at 30- and 15-fold lower concentrations than 25(OH)D_3_ [20]. However, 1,20(OH)_2_D_3_ remained undetectable in human serum, suggesting that circulating 20(OH)D_3_ is not a substrate of renal 1α-hydroxylation [20]. This is consistent with the low affinity of 1α-hydroxylase for 20(OH)D [22]. In contrast, the trihydroxylated metabolites 1,20,24(OH)_3_D_3_, 1,20,25(OH)_3_D_3_, and 1,20,26(OH)_3_D_3_ are found in adrenal extracts, suggesting that 1α-hydroxylation of CYP11A1-produced 20(OH)D_3_ may occur locally in tissues also expressing CYP27B1 such as the adrenal glands, possibly after metabolization by CYP24A1 [20].

The main endocrine regulators of 1α– and 24-hydroxylase expression in the kidney are PTH, FGF23, and 1,25(OH)_2_D. All these endocrine regulatory factors impact on renal 1α– and 24-hydroxylase expression in a reciprocal manner [23]. PTH stimulates the expression of 1α–hydroxylase, and suppresses that of 24-hydroxylase. On the other hand, FGF23 and 1,25(OH)_2_D suppress 1α–hydroxylase, while at the same time stimulating 24-hydroxylase expression [17]. Hence, PTH augments renal production of 1,25(OH)_2_D, whereas FGF23 and 1,25(OH)_2_D suppress it. 

## 3. The Parathyroid-Kidney Axis in the Regulation of Vitamin D Metabolism

**Figure 1:**
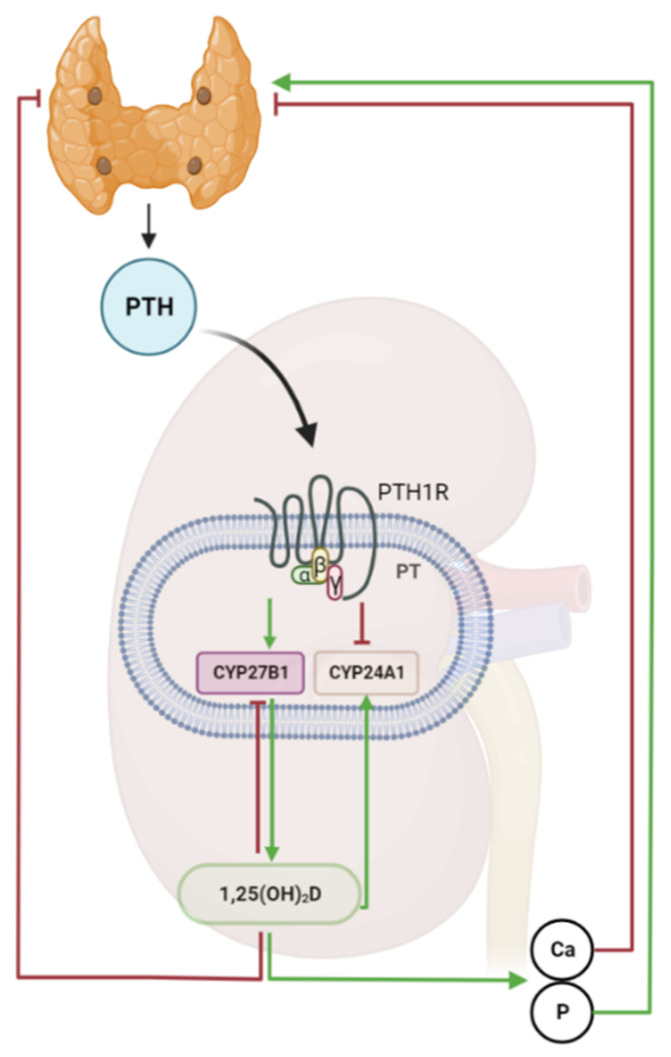
**Parathyroid-kidney axis.** Parathyroid hormone (PTH) secretion from the parathyroid gland is increased in response to a decrease in ionized plasma calcium (Ca) or to an increase in the blood concentration of inorganic phosphate (P). PTH binds to the parathyroid hormone-1 receptor (PTH1R) in proximal tubules (PT) of the kidney, promoting 1,25(OH)_2_D production by upregulating 1α-hydroxylase (*Cyp27B1*) and suppressing 24-hydroxylase (*Cyp24A1*) expression. As part of a feedback inhibition, 1,25(OH)_2_D downregulates its own production by stimulating *Cyp24A1* and inhibiting *Cyp27B1* expression in the kidney. Circulating 1,25(OH)_2_D signals back to the parathyroid gland, where it inhibits PTH transcription and secretion. Arrows indicate the direction and nature of regulation (green, stimulation; red, inhibition). Created with BioRender.com.(Biorender 2022, Toronto, Ontario, Canada).

As mentioned above, it was discovered in the 1970s that 1α,25(OH)_2_D production in the kidney is regulated by PTH, establishing a novel feedback loop between kidney and parathyroid gland [5,6,7,8]. PTH is an 84-amino acid peptide hormone secreted by chief cells of the parathyroid gland in response to changes in the concentration of ionized blood calcium [24]. PTH acts through the parathyroid receptor 1 (PTHR1), a G-protein coupled receptor. All actions of PTH increase blood calcium: PTH signaling increases osteoclast activity, thereby promoting Ca release from the bone [25], favors calcium retention by enhancing Ca reabsorption in the thick ascending limb and in distal convoluted tubule of the kidney [26], and stimulates 1,25(OH)_2_D synthesis in proximal renal tubules, thereby indirectly increasing Ca absorption from the intestines [27]. In turn, normalization of blood calcium levels and increased circulating 1,25(OH)_2_D reduce PTH secretion. Hence, 1,25(OH)_2_D and PTH form a tightly controlled feedback loop in which PTH stimulates 1,25(OH)_2_D synthesis, whereas 1,25(OH)_2_D inhibits PTH secretion (Figure 1). Apart from its effects on calcium metabolism, PTH has a phosphaturic action in the kidney, inhibiting phosphate reabsorption in proximal tubules by downregulating the sodium-phosphate cotransporters NaPi2a and NaPi2c [28]. Therefore, PTH also acts as a phosphate-lowering hormone.

PTH enhances renal 1,25(OH)_2_D production by stimulating the expression of the CYP27B1 enzyme responsible for biosynthesis of active vitamin D, but also by downregulating the expression of CYP24A1, thereby inhibiting vitamin D catabolism [25]. Meyer and coworkers have provided important insights into the mechanisms of how PTH signaling controls *Cyp27b1* transcription in the kidney, using ChIP-Seq analysis [29,30]. The latter authors identified a kidney-specific regulatory region located in an intron of the adjacent *Mettl1* gene that is crucial for the regulation of *Cyp27b1* expression by PTH [29]. Mice with a deletion of the enhancer in the *Mettl1* gene showed a global *Cyp27b1* knockout phenotype with profoundly reduced basal *Cyp27b1* expression, distinctly reduced circulating 1,25(OH)_2_D concentrations, upregulated PTH, and loss of the PTH-mediated induction of *Cyp27b1* [29]. Furthermore, PTH treatment of mice induced a rapid recruitment of the phosphorylated transcription factor CREB to the enhancer region in the *Mettl1* gene in the kidney [30]. ChIP-Seq analysis also revealed that the PTH-induced suppression of the *Cyp24a1* gene in the kidney is mediated through a kidney-specific regulatory region located downstream of the *Cyp24a1* gene [30,31]. Deletion of this regulatory region blunted the suppressive effect of PTH on *Cyp24a1* transcription in vivo [31]. 

As part of a negative feedback mechanism, vitamin D signaling suppresses PTH transcription and secretion as evidenced by a large amount of data from in vivo and in vitro experiments as well as from clinical studies. Incubation of isolated bovine parathyroid cells with 1,25(OH)_2_D for 48 h caused a reduction of PTH mRNA levels to 50% of control values [32]. This was confirmed in mouse parathyroid explants where treatment with vitamin D metabolites reduced PTH secretion [33]. In rats, injection of 1,25(OH)_2_D also decreased PTH gene transcription dose- and time-dependently [34]. This reduction was associated with an upregulation of VDR mRNA levels in the parathyroid gland. Studies in mice lacking VDR have shown that 1,25(OH)_2_D requires the VDR to exert its suppressive effects on *PTH* gene transcription. Administration of 1,25(OH)_2_D had no effect on *PTH* transcription in global VDR knockout mice, supporting the notion that the VDR is required for modulation of PTH by vitamin D signaling. In addition, global deletion of the VDR results in elevated PTH mRNA abundance and profoundly increased PTH serum levels [35]. However, because of the ubiquitous expression of the VDR and the perturbation of mineral metabolism in these mice, it is difficult to distinguish if these effects are due to severe hypocalcemia or if they are indeed due to lack of the VDR in the parathyroid. More insights into this matter were gained when mice with a specific deletion of the VDR in the parathyroid gland were examined. In contrast to global VDR knockout mice, parathyroid-specific conditional VDR knockout mice did not show elevated *PTH* mRNA levels, but presented with moderately increased serum PTH levels [35]. The latter finding was explained by a downregulation of the calcium-sensing receptor in parathyroid-specific VDR knockout mice [35]. In agreement with the latter findings, VDR mutants on a calcium and phosphorus-enriched rescue diet were protected against secondary hyperparathyroidism (sHPT), and a switch of global VDR mutants on rescue diet to a calcium-reduced challenge diet led to severe sHPT associated with hypertrophy and hyperplasia of parathyroid glands, together with profound bone loss. Notably, sHPT was fully corrected by switching VDR mutant mice on a challenge diet back to the rescue diet, suggesting that signaling by the calcium-sensing receptor regulates chief cell function in the absence of signaling through the VDR [36].

In conclusion, there is ample evidence that active vitamin D analogs suppress PTH transcription and secretion in a VDR-dependent manner as part of a pharmacological effect. However, the suppressive effect of vitamin D signaling on *PTH* gene transcription in vivo is dispensable under physiological conditions, suggesting that VDR signaling can certainly suppress PTH secretion, but that the main regulation under normal conditions occurs via ionized blood calcium. 

The exact mechanisms by which active vitamin D analogs modulate *PTH* gene transcription is still controversial. Different mechanisms have been proposed. Mackey et al. demonstrated that the VDR binds directly to a vitamin D response element in the promoter of the human *PTH* gene, independent of the RXR [37]. In the rat *PTH* gene promoter, it was found that VDR/RXR heterodimers bind to a negative, DR3-type element [38]. Another scenario suggests that the liganded VDR does not bind to the *PTH* gene promoter, but rather binds to and inactivates the vitamin D interacting receptor (VDIR), a positive effector of *PTH* gene transcription [39,40]. 

Interestingly, the parathyroid gland not only expresses VDR and 24-hydroxylase, but also 1α-hydroxylase [41]. Circulating 25(OH)D can indeed suppress PTH secretion, but the conversion rate is low, and 25(OH)D has much lower potency when compared to 1,25(OH)_2_D, correlating with its much lower affinity for the VDR [25]. The identification of 1α-hydroxylase in the parathyroid gland suggests that local production of 1,25(OH)_2_D might also influence PTH secretion. However, it remains to be elucidated if local conversion of 25(OH)D into 1,25(OH)_2_D is able to significantly modulate PTH secretion, and if expression of 1α-hydroxylase in the parathyroid gland undergoes any physiological regulation. 

The importance of the vitamin D–PTH axis can be clearly seen in chronic kidney disease (CKD). In patients with CKD, renal 1,25(OH)_2_D synthesis declines as kidney function deteriorates. As a consequence, hypocalcemia and sHPT develop [42]. Therefore, active vitamin D analogs are administered routinely with the aim to normalize PTH levels. However, although the treatment has been shown to be beneficial in terms of reducing PTH levels by 40 to 60% in different randomized control trials, development of hypercalcemia is a possible adverse effect that needs to be closely monitored [43]. It is important to note in this context that over-suppression of PTH by vitamin D analogs is a risk factor for adynamic bone disease in CKD patients [44]. Another potential pitfall of this treatment strategy is that administration of vitamin D analogs tends to become less effective over time in about 20–30% of individuals [45]. Interestingly, not all vitamin D analogs have the same effect on lowering PTH levels, and their potential to induce hypercalcemia is different [45]. Larger randomized controlled trials are necessary to determine whether one agent is superior to the other in terms of the balance between beneficial and untoward treatment effects, in order to optimize treatment strategies in these patients. 

## 4. The Bone-Kidney Axis in the Regulation of Vitamin D Metabolism

The discovery that putative gain-of-function mutations in the *FGF23* gene are the cause of the inherited renal phosphate-wasting disease autosomal dominant hypophosphatemic rickets in the year 2000 heralded a new era in our understanding of vitamin D metabolism [46]. This discovery led to the subsequent unfolding of a previously unrecognized feedback system between bone and kidney, the bone-kidney axis, in the regulation of vitamin D metabolism. Soon after the initial description of FGF23 as a putative phosphaturic hormone [46] it became evident that FGF23 is also a powerful regulator of vitamin D metabolism. When Shimada and coworkers injected recombinant FGF23 into mice they observed not only hypophosphatemia, but also a distinct suppression of renal 1α-hydroxylase (*CYP27B1*) mRNA expression [47]. This finding inaugurated the link between FGF23 and vitamin D metabolism.

FGF23 belongs to the group of endocrine fibroblast growth factors (FGFs), and is mainly produced by osteoblasts and osteocytes in bone [48,49]. During the secretion process, a part of the intact protein is cleaved by proteases such as FURIN into N- and C-terminal fragments at a conserved cleavage site [50]. The intact form of FGF23 is a 32 kDa glycoprotein circulating in the bloodstream. Only the intact form of FGF23 can induce signaling through a receptor complex consisting of FGF receptors (FGFR) and the co-receptor αKlotho [51,52]. FGFR1c is the main FGFR mediating FGF23 signaling under normal conditions [51,53]. The c isoform of FGFR1 is generated by alternative splicing of *Fgfr1* mRNA [53,54].

The secretion of bioactive, intact FGF23 in bone is regulated at the transcriptional and posttranscriptional level by factors such as 1,25(OH)_2_D, PTH, phosphate, iron status, and pro-inflammatory cytokines [55]. Among these factors, 1,25(OH)_2_D is probably the most robust stimulator of FGF23 secretion as evidenced by data from in vivo and in vitro experiments. Treatment of cultured osteoblast-like cells with 1,25(OH)_2_D results in a dose- and time-dependent increase in *Fgf23* transcription [56,57] Similarly, injection of mice with 1,25(OH)_2_D causes a VDR-dependent rise in circulating intact Fgf23 [58,59] In addition, the blood levels of intact Fgf23 are lower in *VDR*- and 1α-hydroxylase-deficient mice than those in WT mice, showing that intact vitamin D signaling is a physiological regulator of Fgf23 secretion [58,59]. Genome-wide association studies in a large number of subjects reported that a single nucleotide polymorphism lying upstream of the *CYP24A1* gene was the strongest predictor of the blood concentrations of FGF23, confirming the importance of the vitamin D hormonal system for the regulation of circulating FGF23 levels in humans [60].

Although it is clear that 1,25(OH)_2_D is a transcriptional regulator of the *Fgf23* gene in a VDR-dependent manner, the exact molecular mechanism of this regulation at the genomic level still remains unclear, and there may be species differences. Whereas several functional VDREs were identified in the promoter of the human *FGF23* gene [61], VDREs are absent in the murine *Fgf23* gene promoter [57], and ChIP-Seq analysis in the murine osteocyte-like cell line IDG-SW3 failed to provide evidence for VDR binding to regulatory regions in the *Fgf23* locus [62]. Therefore, at least in mice, the 1,25(OH)_2_D-induced upregulation of *Fgf23* transcription may be mediated indirectly via DNA binding of other transcription factors. The regulatory region responsible for the 1,25(OH)_2_D-induced upregulation of *Fgf23* transcription in mice has been narrowed down to an enhancer region located directly upstream of the *Fgf23* promoter [63,64]. When this enhancer region was deleted in mice, the 1,25(OH)_2_D-mediated upregulation of *Fgf23* transcription was completely lost [64]. However, the specific transcription factor(s) binding to the enhancer remain unknown. 

Secretion of intact FGF23 is also regulated at the posttranslational level in the bone. Intracellular proteolysis of intact FGF23 by FURIN results in the secretion of the biologically inactive N-terminal and C-terminal fragments. Post-translational O-glycosylation at tyrosine 178 by polypeptide N-acetylgalactosaminyltransferase3 (GALNT3) inhibits FURIN-mediated cleavage, thus favoring secretion of intact FGF23. In contrast, phosphorylation at serine 180 (S180) by extracellular kinase family member 20c (FAM20) inhibits glycosylation, thus facilitating FURIN-mediated cleavage [65]. Hence, the posttranslational modification within or near the FURIN-mediated cleavage site is an important determinant of the ratio between intact and cleaved FGF23 secreted by bone cells [50]. It has been shown that PTH injection acutely alters the ratio of intact to cleaved FGF23 in mice [66]. Whether 1,25(OH)_2_D, similar to PTH, may regulate secretion of intact FGF23 not only at the transcriptional, but also at the posttranslational level remains to be elucidated. 

The most important target organ of the endocrine actions of FGF23 is the kidney. FGF23 targets proximal and distal renal tubules by binding to basolateral αKlotho/FGFR1c complexes [67,68]. In proximal renal tubules, FGF23 signaling has a dual action: (i) it downregulates the apical membrane abundance of sodium-phosphate co-transporters, causing reduced transcellular phosphate uptake from the urine and, hence, increased urinary phosphate excretion [69,70,71]; (ii) it suppresses the transcription of 1α-hydroxylase and increases transcription of 24-hydroxylase, thereby reducing renal production of 1,25(OH)_2_D [71]. In distal renal tubules, FGF23 signaling causes enhanced reabsorption of calcium and sodium by upregulating the apical membrane abundance of the epithelial calcium channel TRPV5 and of the sodium-chloride cotransporter NCC [67,72].

There is compelling evidence from animal experiments and rare genetic disorders in humans that intact FGF23 signaling is essential for the endocrine control of vitamin D metabolism in the kidney. In humans, loss-of-function mutations in *FGF23* or *αKLOTHO* are associated with elevated circulating 1,25(OH)_2_D levels and subsequent hypercalcemia and hyperphosphatemia, leading to progressive soft tissue calcifications [73,74,75]. Similarly, knockout of the *Fgf23* of *αKlotho* genes in mice leads to early lethality caused by unleashed production of 1,25(OH)_2_D, hypercalcemia, hyperphosphatemia, and ectopic calcifications, a phenotype that can be rescued by concomitant ablation of vitamin D signaling [76,77,78,79,80,81,82,83,84]. 

Despite the strong evidence from in vivo studies, the molecular reason for the complete failure of the physiological regulation of renal 1α- and 24-hydroxylases in the absence of FGF23 signaling is still not entirely clear. What do we know about the signaling cascade involved? Based on the similarity of the phenotypes of *αKlotho*^-/-^ and *Fgf23^-/-^* mice, it is clear that the FGF23-mediated suppression of 1α-hydroxylase transcription in proximal tubular epithelium is an *α*Klotho-dependent process [51]. In addition, specific deletion of *Fgfr1* in proximal renal tubules has been shown to blunt the FGF23-induced suppression of circulating 1,25(OH)_2_D levels in vivo [85]. Therefore, at least under physiological conditions, the receptor for FGF23 at the cell membrane of proximal tubules is the FGFR1c/*α*Klotho receptor complex. At high circulating concentrations of FGF23 such as those found in *Hyp* mice, a model of excessive endogenous FGF23 secretion, FGFR3 and 4 may also play some role in the FGF23-mediated regulation of renal vitamin D metabolism, because ablation of *Fgfr3* and *Fgfr4* increases 1,25(OH)_2_D levels in *Hyp* mice [86]. FGFRs are tyrosine kinase receptors, and ligand binding results in dimerization and subsequent activation of intracellular phosphorylation cascades [54]. It is well known that the FGF23-mediated regulation of phosphate transport in proximal tubular epithelium involves activation of extracellular signal-regulated kinase-1/2 (ERK1/2) [68]. Activation of ERK1/2 is also one of the first steps in the control by renal 1α- and 24-hydroxylases by FGF23 signaling, as evidenced by the fact that pharmacological ERK1/2 inhibition increases serum 1,25(OH)_2_D levels in *Hyp* mice [87,88]. However, the signaling pathway downstream of ERK1/2 remains unknown. 

At the genomic level, the seminal studies of Meyer and coworkers have shed additional light on the gene regulatory networks involved in the control of 1α- and 24-hydroxylase transcription by FGF23 signaling [29,89]. ChIP-Seq experiments revealed that the kidney-specific regulatory elements leading to suppression of renal 1α-hydroxylase transcription after FGF23 injection in mice are located in introns of a neighboring gene, the *Mettl21b* gene [29]. The functional significance of these kidney specific enhancer sites were demonstrated by the fact that deletion of these sites completely ablated the FGF23-induced suppression of 1α-hydroxylase gene transcription in vivo [29]. However, the specific transcription factors binding to these regulatory regions remain to be elucidated. In addition, it is unclear why simultaneous deletion of all three FGF23-regulated enhancer sites in the *Mettl21b* gene identified by ChIP-Seq experiments did not result in uncontrolled 1,25(OH)_2_D production and a phenotype similar to *Fgf23* or *αKlotho* deficient mice [29]. A potential explanation for this discrepancy is that FGF23 signaling has reciprocal effects on 1α- and 24-hydroxylase transcription in the kidney, at the same time suppressing 1α-hydroxylase while upregulating 24-hydroxylase transcription [71]. Therefore, the phenotype of *Fgf23* or *αKlotho* deficient mice may only develop in the absence of this dual effect on vitamin D metabolism, affecting both synthesis and degradation of 1,25(OH)_2_D. 

It was previously thought that the regulation of 24-hydroxylase transcription by FGF23 is mediated indirectly through the VDR, because treatment of global VDR knockout mice with recombinant FGF23 suppressed 1α-hydroxylase, but did not upregulate 24-hydroxylase transcription [89]. Moreover, injection of recombinant FGF23 failed to induce 24-hydroxylase transcription in kidney-specific 1α-hydroxylase knockout mice, unable to produce 1,25(OH)_2_D in the kidney [29]. However, it is unclear whether one can infer from the latter experiments that the FGF23-mediated regulation of 24-hydroxylase is VDR-dependent, because both models are characterized by a profound downregulation of renal 24-hydroxylase expression, potentially masking acute effects of FGF23 treatment. Strong evidence in favor of a VDR independent regulation of renal 24-hydroxylase by FGF23 comes from experiments in which an enhancer region downstream of the 24-hydroxylase gene, previously identified by ChIP-Seq experiments, was deleted in mice. In contrast to the 1,25(OH)_2_D-mediated induction of 24-hydroxylase, the FGF23-mediated induction of 24-hydroxylase expression was blunted in these mice, showing that FGF23 and 1,25(OH)_2_D have independent effects on 24-hydroxylase expression in vivo [31]. However, the exact mechanisms by which FGF23 signaling regulates renal 24-hydroxylase are still unknown. 

**Figure 2:**
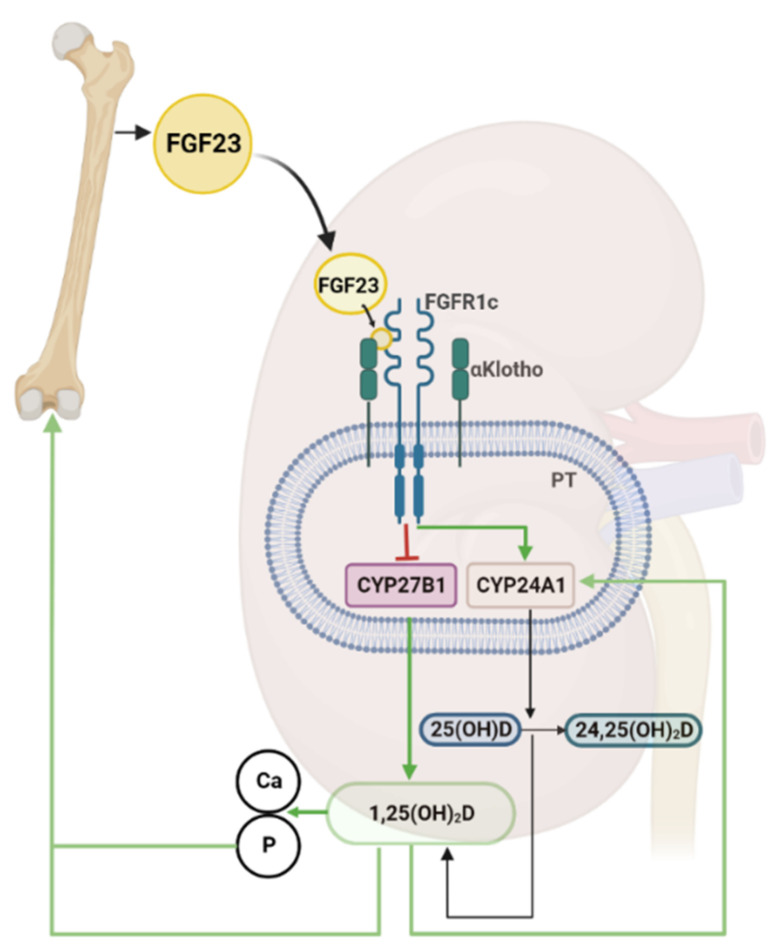
**Bone-kidney axis.** Fibroblast growth factor 23 (FGF23) is mainly produced in bone under physiological conditions. The active vitamin D hormone (1,25(OH)_2_D) and phosphate (P) stimulate FGF23 secretion from bone. Circulating FGF23 binds to a receptor complex consisting of FGF receptor-1c (FGFR1c) and of the co-receptor αKlotho in proximal tubules (PT) of the kidney. FGF23 signaling inhibits transcription of 1α-hydroxylase (*CYP27B1*), the key enzyme for vitamin D synthesis, while stimulating transcription of 24-hydroxylase (*CYP24A1*), the main enzyme responsible for vitamin D catabolism. 25-hydroxyvitamin D (25(OH)D) produced in the liver and circulating in the bloodstream is converted in the kidney into 1,25(OH)_2_D by CYP27B1, or into 24,25(OH)_2_D by CYP24A1. 1,25(OH)_2_D stimulates intestinal absorption of calcium (Ca) and P, thereby increasing the blood concentrations of both minerals. Arrows indicate the direction and nature of regulation (green, stimulation; red, inhibition). Created with BioRender.com (Biorender 2022, Toronto, Ontario, Canada).

Taken together, a paradigm has evolved during the past 20 years in which FGF23 and 1,25(OH)_2_D represent the endocrine signal molecules linking bone and kidney in the regulation of vitamin D metabolism. In this negative feedback system, bone-derived FGF23 suppresses 1,25(OH)_2_D production in the kidney, whereas 1,25(OH)_2_D increases FGF23 secretion in bone (Figure 2). The pathophysiological implications of this feedback system are manifold. In diseases characterized by excessive FGF23 secretion such as X-linked hypophosphatemia or chronic kidney disease, enhanced FGF23 signaling suppresses 1,25(OH)_2_D production in the kidney [90]. However, it is important to consider that treatment with active vitamin D analogs may increase circulating intact FGF23, which in clinical situations with already elevated levels of circulating FGF23 is increasingly recognized as an untoward side effect of the treatment due to the potential negative cardiovascular effects of FGF23 [91]. 

## 5. Vitamin D and the Renin-Angiotensin-Aldosterone System (RAAS)

The most important physiological role of vitamin D is its function in the maintenance of mineral homeostasis. However, in the 1980s seasonal variations in the incidence of cardiovascular diseases were for the first time attributed to seasonal variations in vitamin D status [92]. Together with the findings that the VDR is expressed in cardiovascular tissues such as cardiomyocytes, endothelial and vascular smooth muscle cells, this seminal discovery suggested that vitamin D signaling may have a role in the cardiovascular system [93,94,95,96]. Indeed, observational studies in humans have found an association between vitamin D deficiency and hypertension, left ventricular hypertrophy (LVH) and heart failure [97,98,99,100], and experimental studies in mice and rats provided evidence for a role of vitamin D signaling in the regulation of cardiovascular function. One of the most important regulatory systems of hemodynamics is the RAAS. Juxtaglomerular cells in afferent arterioles in the kidneys store and secrete renin [101]. The main role of renin is to cleave the liver-derived plasma protein angiotensinogen to angiotensin I. Angiotensin I is physiologically inactive and needs to be metabolized to angiotensin II (AngII), which then binds to angiotensin II type I (AT1) and angiotensin II type II (AT2) receptors to exert its effects [101]. The cleavage of angiotensin I to angiotensin II is mediated by angiotensin-converting-enzyme (ACE1) present on vascular endothelial cells mainly in lungs and kidneys. Angiotensin II is a major regulator of peripheral vascular tone, and increases sodium reabsorption from the kidney. Furthermore, it stimulates release of aldosterone from the zona glomerulosa in the adrenal cortex. Aldosterone binds to mineralocorticoid receptors and promotes sodium and water reabsorption in the epithelial cells of the distal convoluted tubule and collecting duct, thereby increasing blood volume and subsequently elevating arterial blood pressure [101,102]. 

In a seminal study published in 2002, Li and coworkers reported that mice with a global VDR deletion were characterized by increased blood pressure, cardiac hypertrophy, increased renin mRNA and protein levels, as well as elevated plasma angiotensin II production [9]. The angiotensinogen levels in the liver were unchanged when compared to WT mice, suggesting that the increase in plasma AngII levels was due to renin over-activity. Although the latter study for the first time established a potential link between vitamin D signaling and the RAAS, a major caveat in that study was that global VDR knockout mice on a normal diet present with hypocalcemia and secondary hyperparathyroidism, both of which can individually influence the cardiovascular outcomes observed. The authors aimed to address this by feeding a calcium- and phosphorus-enriched rescue diet, and by examining mice at 20 days of age with the rationale that hypocalcemia in VDR knockout mice is not yet developed at this early stage. Interestingly, they found that the dietary treatment for five weeks normalized calcium levels in VDR knockout mice, but the mice still showed increased renin expression. However, the rescue diet did not normalize serum PTH, and PTH itself can stimulate the RAAS [9]. Therefore, based on the study by Li and coworkers [9], it was not entirely clear whether the VDR functions as an endocrine suppressor of renin biosynthesis. A later proposed mechanism by which 1,25(OH)_2_D could suppress renin gene transcription is by blocking the activity of the cyclic AMP response element in the renin gene promoter [103]. A recent study in VDR knock-out mice fed the rescue diet found an increase of almost 50% in renin mRNA expression in the kidney, as well as elevated serum renin levels when compared to controls on normal or rescue diet. Interestingly, renin activity and thereby the ability to generate angiotensin I was lower in knock-out mice and there were no significant changes in any of the cardiovascular parameters measured [104]. On the other hand, Jia and coworkers found that *VDR* deficiency in 8 week-old mice increased blood pressure by elevating oxidative stress factors and RAAS activity [105]. However, the authors did not report data on mineral metabolism or PTH levels in these mice. 

Zhou and coworkers utilized 1α-hydroxylase knock-out mice to investigate if the cardiovascular effect of 1,25(OH)_2_D is dependent on calcium and/or phosphorus. 1α-hydroxylase knockout mice have a similar phenotype compared with global VDR knockout mice, making them a useful tool to investigate the role of the VDR in hypertension [106]. Similar to VDR mutant mice, 1α-hydroxylase knockout mice also presented with hypertension and cardiac hypertrophy that was associated with upregulation of the RAAS in cardiac and renal tissue [106]. Treatment of these mice with 1,25(OH)_2_D suppressed RAAS activity and reversed the phenotype [106]. Similar to the study by Jia and coworkers, the authors did not report PTH levels, making it hard to dismiss the role of PTH in mediating cardiovascular changes. 

When we analyzed aged global VDR mutant mice maintained on rescue diet since weaning, we also found increased systolic blood pressure and impaired systolic and diastolic function in 9-month-old VDR knockout mice [107]. However, these changes were due to a lower bioavailability of the vasodilator nitric oxide (NO), leading to endothelial dysfunction, increased arterial stiffness, structural remodeling of the aorta, and impaired systolic and diastolic heart function in 9-month-old mice. Interestingly, kidney renin mRNA levels, urinary aldosterone and plasma renin activity were not different between WT and VDR knockout mice fed the rescue diet, suggesting that the cardiovascular changes induced by the lack of VDR signaling were RAAS-independent. On the other hand, an increase in renin mRNA expression and serum aldosterone levels were observed in global VDR knockout mice fed the normal diet, supporting the notion that secondary hyperparathyroidism might be responsible for RAAS activation and subsequent hemodynamic changes in mice lacking VDR [107].

Mice lacking VDR specifically in the endothelium were able to shed more light on the question whether hypertension in global VDR knockout mice is caused by RAAS activation or is independent of RAAS. These animals had no changes in mineral metabolism, and thereby serve as a better model to test tissue-specific effects of vitamin D [108]. At baseline, the cardiovascular phenotype of 12-week-old endothelium-specific VDR knockout mice was comparable to that of WT mice. However, mice with VDR deletion in the endothelium were more susceptible to treatment with low doses of Ang II as evidenced by increases in systolic, diastolic, and mean arterial pressure when compared to WT mice [108]. Furthermore, in the absence of endothelial VDR, acetylcholine-induced aortic relaxation was significantly impaired, and aortic mRNA and protein abundance of eNOS was reduced, suggesting that the VDR has a protective role in regulating vascular tone and that the changes observed are independent of RAAS [108].

Interestingly, observational studies support an inverse relationship between circulating vitamin D levels and RAAS in hypertensive and non-hypertensive subjects [109,110,111,112]. This was confirmed in a small open-label study where daily treatment of vitamin D deficient, hypertensive individuals with 15,000 IU vitamin D for 4 weeks increased 25(OH)D and 1,25(OH)_2_D, decreased PTH levels, reduced supine mean arterial pressure (MAP) and reduced kidney specific RAAS activation [113]. However, randomized controlled trials investigating the interaction between vitamin D and RAAS are scarce. In the study by McMullan and coworkers, supplementation with 50,000 IU vitamin D per week over eight weeks increased 25(OH)D levels, but neither affected blood pressure nor elements of the RAAS [114]. However, the failure to find an effect of vitamin D supplementation on RAAS in the latter study may be due to the fact that normotensive subjects were investigated, and that the subjects were vitamin D sufficient and that the dosage of vitamin D administered was lower [114]. Similarly, in recent large, randomized controlled trials such as VIDA and VITAL with over 5000 and 25,000 vitamin D sufficient subjects, respectively, no beneficial effects of vitamin D supplementation on primary and secondary cardiovascular endpoints were found [115,116]. Unfortunately, the latter trials did not include measurements of RAAS activity as endpoints. Whether the effects would be similar in vitamin D deficient individuals remains to be investigated.

ACE2 enzyme is a more recently discovered homologue of ACE1. In contrast to ACE1, ACE2 is not as ubiquitously expressed, and is found mainly in the kidneys, heart, and testis [117]. While ACE1 produces Ang II, ACE2 converts angiotensin II (Ang II) into angiotensin 1–7 (Ang 1–7), binds to Mas receptors (MasR), and has a powerful vasodilatory, antifibrotic, antiarrhythmic, and antihypertensive effect [118]. Therefore, ACE2 acts as a negative regulator of the RAAS and together with ACE1 participates in maintaining RAAS homeostasis and blood pressure. It has been reported that vitamin D signaling induces the ACE2/Ang-(1-7)/MasR axis activity, and thereby has a protective role in cardiovascular and pulmonary diseases [119]. Shedding of ACE2 by disintegrin and metalloproteinase domain 17 (ADAM17) from the membrane results in production of soluble ACE2 (sACE2). Interestingly, sACE2 appears to be a biomarker in patients with heart failure, reflecting increased ACE activity [120]. Furthermore, administration of calcitriol to rats with LPS-induced acute lung injury increased ACE2 mRNA expression, and had a positive effect on clinical manifestations and pathological changes in these animals [121,122]. Mice lacking VDR presented with a more severe acute lung injury and higher mortality when compared to WT controls, as a result of excessive induction of angiopoietin-2 and angiotensin II, suggesting that VDR signaling regulates both the angiopoietin-2 and the RAAS pathways [123]. Calcitriol and paracalcitriol decreased ACE1 concentration, ACE1/ACE2 ratio, reduced oxidative stress, and exerted renoprotective effects in diabetic rats, additionally supporting the protective effect of vitamin D [124,125]. It is tempting to speculate that some of the discrepancies observed in animal studies dealing with the effects of vitamin D signaling on the RAAS may be resolved by examining the ACE2/Ang-(1-7)/MasR axis. 

While the regulation of RAAS by vitamin D has been extensively studied, the data on modulation of vitamin D metabolism by RAAS is scarce. Currently, there is no evidence that RAAS can directly influence 1α-hydroxylase activity or VDR expression. However, indirect effects of the RAAS on vitamin D metabolism are conceivable, because impacts of RAAS components on αKlotho and FGF23 have been reported. Notably, in several animal models characterized by RAAS activation there is a clear downregulation of αKlotho abundance in the kidney [126,127,128]. Furthermore, long-term angiotensin II infusion also downregulated renal αKlotho expression at mRNA and protein level [129]. This mechanism is thought to be AT1-dependent, because administration of losartan completely abolished the effect. Several other mechanisms of αKlotho regulation, besides the direct action on AT1 receptor, by Ang II have been proposed. It is known that oxidative stress can downregulate αKlotho expression [130]. Oral administration of a free radical scavenger in rats suppressed the downregulation of αKlotho, supporting the hypothesis that an increase in systemic oxidative stress modulates αKlotho expression [128]. There is accumulating evidence that Ang II stimulates formation of reactive oxygen species (ROS) through the NADPH oxidase system [131,132,133]. Indeed, Ang II receptor blockade reduced oxidative stress in vivo, suggesting that Ang II can modulate Klotho expression by influencing the formation of ROS [134]. Furthermore, Ang II can downregulate Klotho by upregulating TACE, a TNFα-converting enzyme, thereby identifying TACE inhibitors as a new potential therapeutic target in patients suffering from diseases characterized by low Klotho abundance in the kidney [135,136,137]. 

As mentioned above, FGF23 requires the presence of its coreceptor αKlotho in the kidney to exert its effects. In proximal renal tubules, FGF23 signaling inhibits 1,25(OH)_2_D synthesis, and stimulates its catabolism by downregulating 1α-hydroxylase and upregulating 24-hydroxylase, respectively. Therefore, the effect of Ang II on αKlotho could be interpreted as an indirect modulation of vitamin D metabolism by influencing the FGF23- αKlotho axis. 

Chronic kidney disease is characterized by low αKlotho and 1,25(OH)_2_D levels and elevated circulating FGF23. High FGF23 levels are associated with left ventricular hypertrophy and heart failure [138]. Furthermore, increased circulating FGF23 additionally exacerbates 1,25(OH)_2_D deficiency, possibly contributing to faster progression of CKD and increased mortality in these patients. Therefore, it seems plausible that targeting Klotho deficiency by blocking RAAS and thus normalizing 1,25(OH)_2_D production may have beneficial effects in patients with CKD in regard to cardiovascular and renal outcomes. Indeed, RAAS inhibition and supplementation with vitamin D analogs are common treatment strategies in patients with CKD [139]. However, they are usually considered separately, and their potential joint effect on the FGF23-αKlotho axis is not well investigated. Adapting the existing treatment to modulate renal αKlotho levels and the FGF23-αKlotho-vitamin D axis could prove to be a more efficient therapeutic option in patients with CKD. 

## 6. Conclusions

The purpose of this review was to highlight the current knowledge about the molecular mechanisms underlying the interaction of vitamin D, FGF23, PTH and RAAS in health and disease. FGF23 is a key regulator of vitamin D metabolism. FGF23 lowers 1,25(OH)_2_D biosynthesis by suppressing 1α-hydroxylase transcription, and increases its degradation by stimulating 24-hydroxylase transcription in proximal tubules of the kidney, thereby downregulating renal 1,25(OH)_2_D production. PTH on the other hand has the opposite effect on vitamin D metabolism: it stimulates 1,25(OH)_2_D synthesis and inhibits its catabolism. Our understanding of the genomic mechanisms involved in the FGF23- and PTH-mediated regulation of vitamin D metabolism in the kidney has increased significantly during recent years. However, some major gaps in our knowledge still remain. For example, the signaling cascades mediating the FGF23-induced regulation of 1α-hydroxylase and 24-hydroxylase transcription in the kidney are still unknown. Furthermore, studies investigating the role of vitamin D in the cardiovascular system and the relationship between vitamin D and the RAAS have provided conflicting evidence. While there are several proposed mechanisms as to how vitamin D signaling can regulate RAAS, the regulation of vitamin D metabolism by RAAS is still an area largely unexplored.
